# TAPping into the treasures of tubulin using novel protein production methods

**DOI:** 10.1042/EBC20180033

**Published:** 2018-11-14

**Authors:** Nuo Yu, Niels Galjart

**Affiliations:** Department of Cell Biology, Erasmus MC, P.O. Box 2040, 3000 CA Rotterdam, The Netherlands

**Keywords:** microtubule, protein-protein interactions, tubulins

## Abstract

Microtubules are cytoskeletal elements with important cellular functions, whose dynamic behaviour and properties are in part regulated by microtubule-associated proteins (MAPs). The building block of microtubules is tubulin, a heterodimer of α- and β-tubulin subunits. Longitudinal interactions between tubulin dimers facilitate a head-to-tail arrangement of dimers into protofilaments, while lateral interactions allow the formation of a hollow microtubule tube that mostly contains 13 protofilaments. Highly homologous α- and β-tubulin isotypes exist, which are encoded by multi-gene families. *In vitro* studies on microtubules and MAPs have largely relied on brain-derived tubulin preparations. However, these consist of an unknown mix of tubulin isotypes with undefined post-translational modifications. This has blocked studies on the functions of tubulin isotypes and the effects of tubulin mutations found in human neurological disorders. Fortunately, various methodologies to produce recombinant mammalian tubulins have become available in the last years, allowing researchers to overcome this barrier. In addition, affinity-based purification of tagged tubulins and identification of tubulin-associated proteins (TAPs) by mass spectrometry has revealed the ‘tubulome’ of mammalian cells. Future experiments with recombinant tubulins should allow a detailed description of how tubulin isotype influences basic microtubule behaviour, and how MAPs and TAPs impinge on tubulin isotypes and microtubule-based processes in different cell types.

## Microtubules, functions and basic properties

Microtubules (MTs) are hollow tubes, which together with actin and intermediate filaments (IFs) form the cytoskeleton of cells. The MT network stretches throughout a cell and contributes to numerous processes, including mitosis, intracellular transport, cell polarity and cell migration. MTs also form the inner core of cilia and flagella, projections from the cell body that serve as signalling and/or locomotion platforms.

MT assembly and disassembly occur by the addition or removal of tubulin at the ends of MTs in complex processes that are still not understood in detail [1]. Tubulin is a heterodimer of two related globular proteins designated α- and β-tubulin. Both subunits bind GTP, however, only GTP in β-tubulin undergoes hydrolysis; this occurs after heterodimer incorporation into MTs. Tubulin dimers assemble in a polarized head-to-tail fashion to form protofilaments via longitudinal interactions, and these fold up through lateral interactions to form a hollow MT with a diameter of 25 nm. Most MTs formed *in vivo* contain 13 protofilaments, whereas *in vitro* the number can fluctuate somewhat. One end of the MT (called the plus end) exposes the β-tubulin subunit of the dimer, whereas the other (the minus end) exposes α-tubulin. Tubulin dimers can incorporate (and disassemble) at both ends; however, in cells the MT minus end is often non-dynamic because it is embedded in a protective structure like the centrosome.

MTs can switch between growing and shrinking phases by undergoing catastrophes (the conversion of MT growth to shrinkage) or rescues (the switch from shrinkage to growth), respectively. As mentioned above, GTP hydrolysis occurs shortly after incorporation of a tubulin dimer into a MT. Thus, the polymerizing MT plus end carries a GTP-tubulin ‘cap’ formed by newly assembled GTP-bound dimers. GTP hydrolysis induces strain in the GDP-tubulin MT lattice [2]. Upon disappearance of the GTP ‘cap’, for example because of a lowered MT polymerization rate, GDP-tubulin becomes exposed at the plus end, and it is thought that the release of strain underlies catastrophe and rapid MT shrinkage. Thus, dynamic MTs are steady state machines switching between growth and shrinkage in a stochastic manner, a behaviour termed dynamic instability [3].

## Tubulin isotypes, biosynthesis and regulation

In mammals, multiple highly homologous α- and β-tubulin isotypes have been identified, which are encoded by small gene families. For example, in humans there are eight α- and nine β-tubulin encoding genes [4] (note that Table 5.1 in this study lists the human and corresponding mouse tubulin-isotype encoding genes). Here, we indicate the genes encoding specific tubulin isotypes using italics and normal lettering (e.g. *TUBB3*), and we use Greek and Roman lettering to indicate tubulin isotype proteins (e.g. βIII).

Biosynthesis of tubulin isotypes is highly regulated at different molecular levels. For example, it has been shown that the application of MT depolymerizing agents such as colchicine or nocodazole, which increase tubulin protein concentration in the cell, leads to a rapid and specific degradation of tubulin-encoding mRNAs [5]. These data suggest the presence of a mechanism in cells that senses the amount of soluble tubulin and buffers cells against excess tubulin via mRNA degradation. In the case of β-tubulin, this auto-regulatory mechanism appears to be dependent on the first four aminoacids (MREI) that are conserved in all β-tubulin isotypes, while α-tubulin-encoding mRNAs are regulated differently [6]. The detailed mechanisms underlying the control of tubulin mRNA stability still need to be elucidated, but it is interesting to note that knockdown of the MT regulators MCAK or KIF18A leads to increased tubulin synthesis [7], indicating that lower tubulin levels, resulting from a higher MT fraction due to increased MT stability, are also sensed by cells.

Biosynthesis of tubulin dimers that are competent to incorporate into MTs occurs through the so-called tubulin-folding pathway, which involves both common and tubulin-specific folding factors [8]. Like many other proteins, nascent tubulin monomers are first bound by common chaperones of the Hsp70 family. Upon completion of translation, the tubulin polypeptides are transferred, with the help of Prefoldin, to cytosolic chaperonins, which facilitate productive folding in an ATP-dependent manner. Interestingly, point mutations in β-tubulin, which impair proper folding, prevent transfer to the tubulin folding cofactors, and instead these mutants are degraded by the proteasome [9]. Thus, besides auto-regulation at the mRNA level, a protein quality control mechanism operates early on in the tubulin-folding pathway.

After folding by chaperonins, quasi-native tubulin monomers form heterodimers with the help of conserved tubulin-specific folding cofactors (TBCA-TBCE). Monomers are first captured by TBCA (in the case of β-tubulin) or TBCB (in the case of α-tubulin). In one view TBCA/β-tubulin and TBCB/α-tubulin then transfer their respective products to two other folding cofactors, i.e. TBCD and TBCE, respectively. These components subsequently form a super complex (TBCD/β/TBCE/α), and entry of TBCC into this complex induces GTP hydrolysis in β-tubulin [8]. This reaction acts as a switch for the release of native α/β-tubulin heterodimers, which become assembly-competent in the cytoplasm by an exchange of GDP for GTP on β-tubulin. Cofactors TBCC, TBCD and TBCE also stimulate GTPase activity of previously formed α/β-tubulin heterodimers, a reaction that is thought to function as quality control mechanism on the soluble tubulin pool.

An alternative view – the ‘cycling catalytic chaperone’ model – holds that TBCD and TBCE form a stable complex with another cofactor called Arl2, a GTPase, and this stable chaperone complex (called TBC-DEG) either co-assembles α-tubulin and β-tubulin that are handed over by TBCB and TBCA or binds soluble tubulin dimers already present in the cytoplasm [10]. Subsequent binding of TBCC would stimulate GTP hydrolysis on both Arl2 and tubulin and thereby control the direction and/or speed of the reaction. The TBC–DEG complex controls biogenesis, quality and degradation of soluble tubulin, thereby maintaining a ‘healthy’ tubulin pool in the cytoplasm, but how the various activities are coordinated remains to be investigated [10].

Tubulin isotypes are expressed in a developmental and tissue-specific manner, suggesting isotype-specific functions in different tissues. This is corroborated by the observation that βI-tubulin, which together with βIII-tubulin has a slightly longer C-terminal tail compared with other β-tubulins, is highly enriched in megakaryocytes and is essential for platelet formation [11]. βIII-tubulin in turn is enriched in neurons, but has also been found to be up-regulated in many cancers, and although recent data suggest otherwise [12], there is considerable evidence to support the view that βIII-tubulin plays a role in the resistance of cancer cells against chemotherapy [13].

The brain consists primarily of two broad classes of cells: neurons and glial cells. After amplification of neuronal precursor cells by multiple rounds of mitosis, neurons migrate away from the proliferative (ventricular) zone to reach their destination in the brain, and differentiate to establish connections [14]. The critical importance of the MT cytoskeleton and tubulin isotypes for correct neuronal proliferation, migration and differentiation is exemplified by the discovery of mutations in various α- and β-tubulin encoding genes, which result in a broad spectrum of neurodevelopmental disorders [4]. The molecular mechanisms underlying these so-called ‘tubulinopathies’ are still unclear, among others because it is not yet known how tubulin isotypes contribute to MT behaviour, what roles MTs have in neurons as they divide, migrate and differentiate in the developing brain, and which processes are affected by tubulin mutations. Recent data point to localized expression of some of the tubulin-encoding mRNAs in the growth cones of neurons [15], adding an extra layer of complexity to the understanding of tubulin isotype function in health and disease.

## Regulation of MT behaviour by MT-associated proteins

After completion of αβ-tubulin synthesis, dimers can self-organize into MTs; however, the kinetic barrier of MT nucleation is high, meaning that the rate at which MTs spontaneously nucleate, and display initial growth, is low [16]. Thus, in cells most MTs are assembled at specific sites, where nucleation and initial elongation of nascent MTs are stimulated by dedicated factors, including the γ-tubulin ring complex that ensures the formation of MTs with 13 protofilaments. The best known cellular MT nucleation site is the centrosome, which consists of two orthogonally arranged MT-based centrioles that are surrounded by an electron-dense matrix, the pericentriolar material (PCM), which nucleates and anchors cytoplasmic MTs during interphase and mitosis. It has recently been proposed that PCM assembly is driven by macromolecular crowding; this would serve to increase the local concentration of tubulin and other proteins, which in turn would underly efficient MT nucleation [17].

Non-centrosomal MT nucleation also occurs in many cells, for example at the Golgi apparatus, where it contributes to directed cell motility [18,19]. MT nucleation at the Golgi is mediated by the Golgi-resident protein GCC185 (or GCC2), which interacts with CLASPs to establish a platform for MT nucleation. RNAi-studies in Drosophila S2 cells have furthermore indicated multiple factors, including Mini-spindles, CLIP-190 and EB1 (Drosophila homologues of XMAP215, CLIP-170 and EB1, respectively, see below), which contribute to acentrosomal, non-Golgi MT nucleation in the cytoplasm [20], but how this type of MT nucleation is established is unclear.

**Figure 1:**
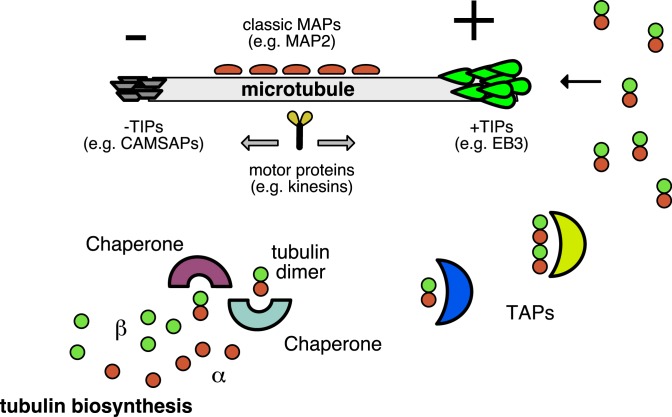
Schematic outline of different classes of MAPs and TAPs α- and β-tubulin isotypes are generated as monomers (α-tubulin: red balls, β-tubulin: green balls). These subunits fold and then they interact to form functional, assembly-competent tubulin dimers with the help of various types of chaperones, which are a prime example of a class of TAPs. Other TAPs are also present in the cellular cytoplasm and bind tubulins for various reasons. Tubulins assemble into MTs. One growing MT is schematically indicated, with its plus (+) and minus (-) ends. MAPs regulate MT behaviour and are often categorized by their mode of binding. Indicated are ‘classic’ MAPs that bind along the MT lattice, +TIPs and -TIPs, which accumulate at growing plus and minus ends, respectively, and motor proteins, which transport cargo along MTs.

Whereas the minus end of a MT is often protected, for example by anchorage to the centrosome, and is therefore not dynamic, the plus end undergoes cycles of polymerization, pauzing, and depolymerization. MT plus- and minus-end properties and the overall cellular organization and behaviour of MTs are modulated by a plethora of microtubule-associated proteins (MAPs), which can be grouped according to their mode of association with MTs (Figure 1). For example, MAP2 and MAP4 are ‘classic’ MAPs that bind along the MT lattice and stabilize MTs [21], proteins such as the CAMSAPs specifically bind to, and stabilize, the minus ends of MTs [22], whereas kinesins and dyneins are motor proteins, which are mainly involved in cargo transport along MTs [23,24]. Yet another interesting group of MT regulators is formed by MT plus-end tracking proteins (+TIPs), of which many have been characterized [25]. These factors accumulate at the ends of growing MTs (Figure 1) and are thereby exquisitely positioned to control MT fate and the interactions of MT plus ends with other structures and organelles. MT plus-end tracking is beautifully captured in fluorescence microscopy-based live imaging experiments, where time-lapse movies of cells expressing fluorescently tagged +TIPs reveal comet-like fluorescent structures that move through cells [26]. While it was clear from the beginning that the fluorescent ‘comets’ represented the ends of growing MTs ‘coated’ with fluorescent +TIPs, understanding how this fascinating behaviour comes about has been difficult. A major mechanism involves recognition of a specific tubulin conformation by the EB family of proteins (EB1-3) [2,27], which have been shown to bind MT plus ends autonomously [28,29]. It should be noted that EB proteins also bind growing MT minus ends, indicating that similar tubulin conformations exist at the ends of growing MT plus and minus ends.

Another factor that binds MT ends autonomously is XMAP215 (also called CKAP5 or ChTOG), a MT polymerase [33] that simultaneously binds tubulin using its five TOG domains and the MT lattice with a basic region, and that positions dimers favourably for incorporation into MTs [34]. High resolution fluorescence microscopy revealed that XMAP215 is positioned more proximally at the MT end as compared with EB1 [35], consistent with a role as MT polymerase. Addition of purified EB1 and XMAP215 together to *in vitro* polymerizing MTs has been shown to increase the speed of MT assembly to levels observed in cells [36], indicating that EB1 and XMAP215 play prominent roles in determining MT growth rates *in vivo*. XMAP215 has also been shown to stimulate MT nucleation and initial growth at the centrosome, and to function together with the γ-tubulin ring complex [37,38]. Thus, XMAP215 is the prototype example of a tubulin-binding +TIP. Interestingly, the capacity of TOG domains to bind tubulin has been exploited by generating an affinity column consisting of bacterially expressed TOG domains from Stu2 (the yeast homologue of XMAP215), covalently coupled to Sepharose [39]. Using this affinity matrix assembly-competent tubulin was isolated from different species.

Virtually all other +TIPs require EB1-3 to bind MT plus ends – a phenomenon called ‘hitch-hiking’. It is for these reasons that the EB proteins have been termed ‘master’ +TIPs. Many of the ‘hitch-hiking’ +TIPs bind EB1-3 using short linear motifs with either SxIP or LxxPTPh as determining sequence [40,41]. The SxIP motif is often embedded in a basic serine-rich region (S/R domain) and basic residues may contribute to MT binding by +TIPs. Mammalian CLASP1 and -2 are examples of such SxIP-type proteins [42]. Whereas only one CLASP1 isoform of ∼160 kDa has been characterized to date (called CLASP1α), alternative promoter usage gives rise to three CLASP2 isoforms of ∼160 kDa (CLASP2α) or ∼140 kDa (CLASP2-β and -γ). CLASP1/2-α isoforms contain three so-called TOG-like (TOGL) domains, one of which (TOGL1) is located at their N-terminus. The shorter isoforms (CLASP2-β and -γ) do not have TOGL1. All isoforms contain two other TOGL domains (TOGL2 and -3), which surround the SxIP motif-containing S/R region, and a C-terminal protein–protein interaction domain, which binds factors such as the +TIPs CLIP-115 and CLIP-170 [42], GCC185 [18], and LL5β [43]. Furthermore, RNA-sequencing of neuronal cell populations has revealed alternative splicing in the *CLASP* genes, resulting in proteins with small internal variations [44]. Interestingly, the TOGL motifs in CLASPs are structurally similar to the TOG domains in XMAP215 [45]. Moreover, fission yeast CLASP (called Cls1p) binds tubulin using its TOGL motifs and promotes MT rescues using bound tubulin, and purified human CLASP2γ was found to bind soluble tubulin [46]. These data suggest that mammalian CLASPs bind tubulin using their TOGL motifs.

It is important to realize that whatever the mode of plus-end association, the binding of +TIPs to MT ends is transient, with association–dissociation occurring on a sub-second time scale [30]. The fluorescent ‘comet’ that appears in live imaging studies therefore represents the hundreds of binding sites for +TIPs at a given MT end [31]. Similar to the GTP ‘cap’, these sites arise shortly after the incorporation of tubulin dimers into the MT lattice and decay in an exponential fashion on a time scale of seconds. Thus, a single +TIP can bind a given site at a MT end multiple times before that site disappears. Since many +TIPs also interact with each other, it has been speculated that +TIP networks at MT ends form conglomerates that stimulate relatively uninterrupted MT growth by acting as ‘MT polymerization chaperones’ [32].

## TAPping into the treasures of tubulin: novel recombinant tubulin production methods

Functional (i.e. assembly-competent) tubulin can be purified from pig or cow brain [47] and is used world-wide in *in vitro* studies of tubulin and MT function. However, brain-derived tubulin preparations are of unknown isotype composition, and since neuronal MTs are quite stable and many of the post-translational modification (PTM) enzymes act on MTs, and not on soluble tubulin [48], brain-derived tubulins are likely to carry a multitude of PTMs. These are primarily located at the C-termini of tubulins and may influence the properties of *in vitro* synthesized dynamic MTs as well as the association of selected MAPs. Using immunoaffinity chromatography, Wilson and colleagues were able to purify αβII, αβIII, and αβIV tubulin isotypes from complex brain tubulin preparations [49]. Videomicroscopy on *in vitro* polymerizing MTs revealed that MTs assembled from αβIII tubulin were more dynamic, displaying higher growth and shrinkage rates and spending less time in the pause state, compared with total brain tubulin. Furthermore, αβIII-derived MTs were less stable [49]. These data showed that β-tubulin isotypes have distinct effects on MTs, and warranted further research into how isotypes influence MT behaviour. However, immunoaffinity chromatography-based approaches require considerable amounts of mono-specific antibodies, which do not exist for most of the tubulin isotypes.

Mammalian tubulins of defined isotype might be produced in a recombinant fashion. Attempts to do this in bacteria or yeast were not successful probably because of the complexity of mammalian tubulin biosynthesis. However, in 2013 assembly-competent human recombinant tubulin was obtained from baculovirus-infected insect cells [50]. In this first study, tubulins were tagged at their C-termini, with a His tag for human αIB (encoded by the *TUBA1B* gene) and a Flag tag for βIII (encoded by *TUBB3*). This strategy was subsequently used to purify recombinant tubulin with point mutations in βIII that cause congenital fibrosis of the extraocular muscles type 3 (CFEOM3) in man [51]. It was shown that disease-causing *TUBB3* mutations severely affected kinesin function.

The baculovirus insect cell strategy [50] and the generation of a TOG-based tubulin affinity matrix [39] have opened up the field of tubulin research. For example, a TEV-protease-cleavable His-tag was added to the C-terminus of human βIII- or βIIB-tubulin, and these tubulins, expressed together with non-tagged human α-tubulin, were purified from insect cells, using a TOG-affinity-column as a last purification step [52,53]. This approach yielded pure and functional human β-tubulin, in dimers with either human or insect α-tubulin. In another approach, an internal His-tag was placed in the acetylation loop of α-tubulin, and βIII-tubulin was tagged with a PreScission protease-cleavable C-terminal FLAG-tag [54]. This yielded pure human recombinant tubulin dimers, in which the only exogenous modifications were the internal His-tag and five amino acids at the C-terminus of β-tubulin (remnants of the PreScission cleavage). In yet another approach, a modified TOG-affinity method was used to purify tubulin from a human embryonic kidney cell line (tsA 201); this yielded a fairly homogeneous tubulin isotype preparation because the tsa 201 cells express only few tubulin genes [55].

Recently, the tubulin production protocol in insect cells was further optimized by co-expressing human αIB-tubulin, tagged at its N-terminus with a TEV-protease-cleavable His-tag and a two amino acid linker, and either βIII- or βIIB-tubulin, which were tagged at their C-termini with a TEV cleavage site followed by a small linker and a Strep II tag [56]. Affinity tag-free pure recombinant human αIB/βIII- or αIB/βIIB-tubulin dimers were obtained in high quantities using affinity columns for the N- and C-terminal tags, followed by TEV cleavage and some additional purification steps. Strikingly, these pure tubulin preparations revealed that variation in β-tubulin isotype leads to *in vitro* assembled MTs with different protofilament numbers. Moreover, consistent with previous results using brain-derived purified αβIII tubulin [49], MTs containing αIB/βIII-tubulin were found to be less stable than those containing αIB/βIIB-tubulin, both to spontaneous disassembly and depolymerization induced by MT regulators [56]. In conclusion, it is now possible to tap into the *in vitro* treasures of tubulin isotypes using defined tubulin preparations. This will increase mechanistic understanding of, for example, the regulation of MT dynamics in specific subcellular regions by tubulin isotype composition, or how tubulin mutations contribute to MT dysfunction in neuronal disorders.

**Figure 2:**
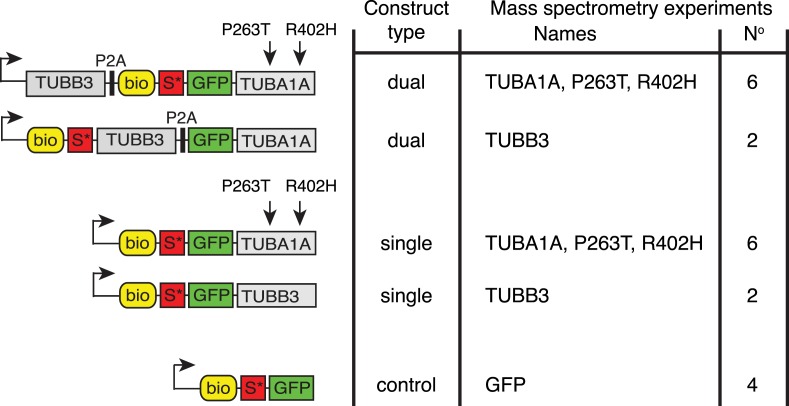
Schematic representation of tubulin expression constructs Constructs named ‘dual’ express two tubulins from one mRNA by virtue of the P2A system. Constructs named ‘single’ express only one tubulin subunit. The right hand of the figure indicates use of the constructs in the mass spectrometry experiments aimed at identifying TAPs.

Insect cells are good vehicles for the purification of recombinant mammalian tubulins and for *in vitro* studies. However, cellular analysis of mammalian tubulins requires other approaches. We reasoned that overexpression of single exogenous α- or β-tubulin subunits in mammalian cells would clog the tubulin-folding pathway because of a subunit imbalance and lead to dysfunction. We therefore designed constructs that allowed co-expression of exogenous α- and β-tubulin subunits at equimolar levels in transfected mammalian cells (Figure 2, see also [57]). In these constructs, the cDNAs encoding human αIA- and βIII-tubulin (*TUBA1A* and *TUBB3*, respectively) were interrupted by a P2A self-cleaving peptide sequence of 22 amino acids that mediates efficient internal ‘cleavage’ of proteins during translation [58]. ‘Cleavage’ is due to pausing of the ribosome, followed by a translation-termination reaction on a codon coding for proline (in the context NPGP) and subsequent downstream (re-)translation [59]. To affinity-purify the tubulins, we tagged αIA- or βIII-tubulin with a small biotinylation sequence, which is recognized by the bacterial BirA enzyme [60], which was co-transfected in cells. We also introduced a Sumo* (or SUMOStar) tertiary recognition sequence of 99 amino acids after the biotin tag. This sequence is recognized by the Sumo* protease, a derivative of Sumo protease [61], which cleaves with high specificity at the C-terminus of the Sumo* sequence. Finally, we tagged α1A- or βIII-tubulin with GFP (Figure 2) to allow fluorescent visualization of recombinant tubulins. We transiently expressed tagged tubulins in HEK293 cells using either the ‘dual’ constructs described above or constructs encoding ‘single’ tagged α- or β-tubulin. Furthermore, we introduced two point mutations into α-tubulin (Figure 2), found in patients with brain malformations [62,63]. Importantly, these point mutations do not compromise the folding of mutant tubulins [64].

Functionality of GFP-tagged α1A-tubulin was shown by purifying proteins from transfected HEK293T cells using streptavidin-coated beads and Sumo* protease, and demonstrating incorporation of purified GFP-tubulin into *in vitro* synthesized MTs [57]. However, we noted that only ∼70 % of the tubulin purified from HEK293T cells was functional because the remainder was associated with chaperones. This is logical since we used transiently transfected cells, in which overexpressed tubulins are constantly synthesized and thus all stages of folding and maturation are expected to be isolated. In addition, we noted that the P2A-based method produced less tubulin compared with insect cell-based systems. Thus, future experiments in mammalian cell systems should be aimed at increasing the amount of functional exogenous tubulin.

## TAPping into the treasures of tubulin: characterization of novel tubulin-associated proteins

While many MAPs have been characterized over the years, a systematic identification of tubulin-associated proteins (TAPs) in cells has not been vigourously pursued. This is odd because known TAPs, such as chaperones, chaperonins, tubulin-folding factors, XMAP215, Stathmin and CRMP2, are all quite important for cells. In one mapping approach, tubulin was coupled to Sepharose and a plant cell lysate was passed over the tubulin affinity column [65]. Over a 100 potential TAPs were identified, the vast majority of which were also found to bind MTs. Plant TAPs were categorized into six groups based on predicted functions, and found to be MAPs, translation factors, RNA-binding proteins, signalling proteins, metabolic enzymes or proteins with other functions [65]. Interestingly, plant CLASP was one of the identified TAPs, suggesting that it binds tubulin. In another approach, a column was made of a monoclonal antibody (Y1/2) that recognizes the C-terminal tyrosine of α-tubulin [66]. Purified tubulin was first passed over this column to generate antibody–tubulin complexes, and subsequently interphase or G2/M HeLa cell lysates were passed over to isolate TAPs binding to the antibody–tubulin complexes and identify them by mass spectrometry. Fourteen potential TAPs were found in the interphase HeLa cell extract, including XMAP215 and several chaperones, whereas ten TAPs were identified in the G2/M extract [66]. The presence of chaperones indicates that the tubulin matrix prepared in this manner contained partly unfolded tubulin dimers, perhaps because antibody binding to the C-terminal tyrosine of α-tubulin caused exposure of tubulin domains and binding by chaperones.

Our approach of expressing tagged tubulins in mammalian cells provided us with an opportunity to perform a comprehensive TAP mapping study and to compare wild-type tubulin with mutant proteins. We therefore purified recombinant tubulins from HEK293T cells using mild washing conditions to retain TAPs [57]. We did not include an ATP wash step, which was shown to significantly reduce co-purification of chaperones with tubulin dimers on the TOG affinity matrix [39]. In order to improve confidence in the HEK293T cell ‘tubulome’, we examined two types of purified material. The first sample was derived from proteins present on streptavidin-coated beads. However, this material also contained endogenously biotinylated factors, for example mitochondrial proteins and contaminants with high affinity for the beads. Hence, in a second sample we investigated proteins that were cleaved from the beads by the Sumo* protease. The underlying thought was that contaminants would not be cleaved off and would therefore not be present in the released fraction.

**Figure 3:**
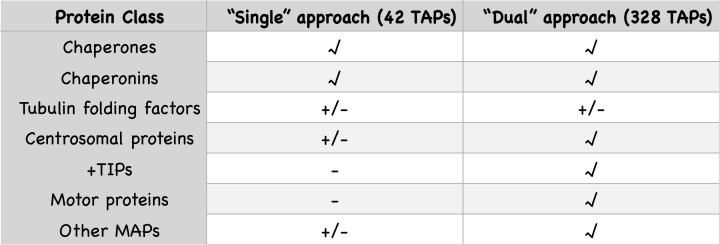
Comparison of mass spectrometry experiments We pulled down biotinylated tubulins from HEK293T cells expressing either ‘single’ or ‘dual’ tubulin constructs. TAPs were co-precipitated and identified using mass spectrometry. The ‘single’ approach yielded 42 potential TAPs, whereas the ‘dual’ approach yielded 328 candidate TAPs (note that these numbers were obtained after filtering of the original mass spectrometry data). Here, we compare co-precipitation of different classes of proteins (V: various proteins of this class are present in the dataset, +/-: only few proteins of this class are present in the dataset, - : no proteins of this class are present in the dataset).

Using this strategy, we performed 20 different mass spectrometry experiments (Figure 2). We indeed found that contaminants such as biotinylated mitochondrial proteins were present in the bead preparations but not in Sumo*-released fractions. Ten of the twenty mass spectrometry experiments (i.e. the ones where we used ‘dual’ constructs) have been published, as these show a more extensive ‘tubulome’ of HEK293T cells [57]. It is nevertheless interesting to compare data of ‘single’ and ‘dual’ approaches (summarized in Figure 3) and to analyse results using the ‘single’ tubulin constructs (shown in Table 1). A comparison reveals first of all that we obtained fewer TAPs co-purifying with ‘single’ expressed tubulins than with ‘dual’ ones (42 versus 328 TAPs, Figure 3). In addition, the majority of TAPs pulled down with the ‘single’ approaches are chaperones/chaperonins (Table 1). Both results are logical, as the ‘single’ overexpressed tubulins are present in excess in the transiently transfected cells; as they cannot dimerize, they remain trapped in the tubulin-folding pathway. One interesting factor, which we observed in pull downs of tagged ‘single’ β-tubulin, but not in other ‘single’ pull downs or in controls, is eukaryotic elongation factor 2 (EEF2, see Table 1). EEF2, which is essential for protein synthesis, is phosphorylated after application of the MT-stabilizing agent taxol, and therefore appears to be involved in the general inhibition of translation that takes place upon taxol treatment [67]. Since EEF2 appears to have a preference for tagged β-tubulin subunits, we wonder whether it is involved in the regulation of the stability of β-tubulin-encoding mRNAs described above.

**Table 1:** Characterization of tubulin-associated proteins

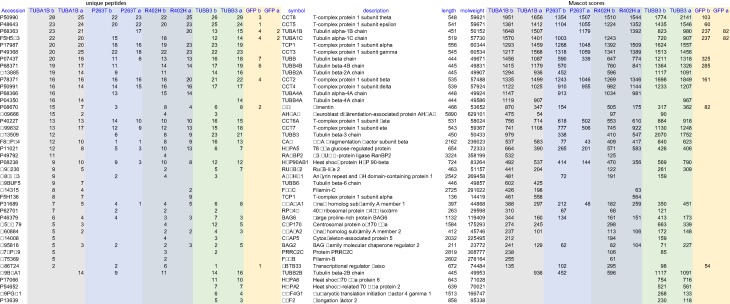

Unique peptides from identified proteins in the paired mass spectrometry analysis are shown in the columns to the left, mascot scores are shown in the columns to the right. TUBA1B: pull-down with tagged wild-type α-tubulin; P263T and R402H: pull-downs with the respective tagged mutant α-tubulins; b: bead fraction; a: fraction released from beads after cleavage with Sumo* protease.

Pull downs using the ‘dual’ constructs revealed many interesting candidate TAPs. Several of these proteins, for example chaperones/chaperonins, tubulin folding factors and XMAP215, are known TAPs. Furthermore, pull downs did not bring down regular MT-binding proteins such as MAP4, KIF5B (conventional kinesin heavy chain) or EB1, despite the fact that RNA-Sequencing revealed abundant expression of the mRNAs encoding these proteins. These data suggest that pull downs were specific for TAPs. However, we did not pull down all known TAPs. For example, Stathmin was lacking from our lists even though the *STMN1* mRNA is highly abundant in HEK293T cells. Thus, ‘tubulome’ mapping studies should be further optimized and ‘tubulomes’ of different cell types should be identified and compared. Stringent filtering of the ‘dual’ list of 328 potential TAPs yielded 45 prominent HEK293T candidates, most of which were novel. RNA-sequencing revealed low-to-moderate expression levels for most of these potential TAPs, considerably lower than the chaperones/chaperonins. Although RNA-Sequencing data cannot be extrapolated to protein levels, they do suggest that many of the TAPs that we pulled down are not abundantly expressed in HEK293T cells. Their identification therefore appears to be due to affinity for the tagged tubulins and not to protein abundance.

We drew four major conclusions from the identification of the HEK293T cell ‘tubulome’ [57]. First, data suggested that TAPs are of particular importance in neurons, as several TAPs (e.g. WDR62, ASPM and KIF2A) have been shown to be mutated in patients with brain malformations. Second, both mutant tubulins interacted less efficiently with a number of TAPs, including centrosomal proteins, +TIPs and GCC185. These results indicate that MT-based processes involving these proteins might be specifically affected in tubulinopathy patients. Third, TAPs in the dataset often interact with other TAPs of the same protein family. For example, CEP97 and CCP110 are two centrosomal TAPs that interact with each other and regulate ciliogenesis [68]. Multiple interactions also exist between the tubulin-binding +TIPs. The finding that the Golgi-associated CLASP-interaction partner GCC185 [18] is also a TAP suggests that CLASPs and GCC185 might both be directly involved in MT nucleation at the Golgi. Thus, tubulin-containing higher order complexes may have particular roles in the regulation of MT nucleation and growth. Finally, since the TOG(L) proteins XMAP215 (CKAP5) and CLASPs were among the most prominent TAPs in the ‘dual’ tubulin pull downs, we concluded from our experiments that the TOG and the TOGL motifs both are potent tubulin binding domains. However, it should be noted that crystallization of TOGL2 and TOGL3 of human CLASP2γ revealed conformations that are suboptimal for binding soluble tubulin [69]. In addition, recent studies showed that none of the TOGL domains (TOGL1-3) from human CLASP2α is able to bind soluble tubulin [70]. By contrast, pull down of full-length His-tagged human CLASP2γ revealed binding to soluble tubulin, and CLASP2γ that was prebound to MTs was able to attract fluorescent tubulin to the MT lattice [46]. We tested CLASP binding to tubulin in co-precipitation studies of YFP-tagged CLASP1α or CLASP2γ with biotinylated tubulin [57]. We found that the longer CLASP1 isoform, CLASP1α, was pulled down more efficiently, suggesting that the TOGL1 motif of CLASP1α enhances tubulin binding. Thus, full-length α-isoforms of the CLASPs appear to bind soluble tubulin, whereas the single TOGL domains, expressed as fusion protein and tested in isolation, do not.

We used fluorescent imaging to examine tubulin incorporation into MTs in ‘dual’ transfected cells. Compared with wild-type tubulin, we found less GFP-tagged mutant tubulin per unit MT length, suggesting a reduced capacity of mutant tubulins to assemble into MTs [57]. Strikingly, when we added the MT-stabilizing agent taxol in a concentration that drives tubulin into MTs and impairs the localization of +TIPs at MT ends, we observed that mutant tubulins were competent to assemble into MTs, consistent with the observation that they fold normally [64]. Since we found that several +TIPs, in particular the CLASPs and CLIP-115, and to a lesser extent XMAP215, bind mutant tubulins less efficiently, we propose that selected +TIP-TAPs form a protein quality control mechanism that operates at MT ends to regulate the incorporation of properly folded tubulin into MTs. This protein quality control mechanism biases against mutant tubulin incorporation and selects in favour of wild-type tubulin. This idea extends the model of +TIPs forming ‘MT polymerization chaperones’ [32] and suggests that tubulin ‘health’ is measured not only at the level of tubulin biosynthesis but also when tubulin is incorporated into MTs. Methods for tubulin purification and TAP identification in mammalian cells can be further optimized, for example, by developing an inducible system with which the expression of exogenous tubulins can be turned on and off at will. This should allow a deeper understanding of tubulin biosynthesis and regulation, and of the regulation of MT-based processes.

## Summary

- Microtubules are cytoskeletal elements with important cellular functions.
- Microtubules are built up of highly homologous tubulin isotypes.
- New protein production approaches have been developed to study tubulins.
- The new methods are revealing tubulin isotype function and cellular tubulomes.

## Abbreviations

EEF2: eukaryotic elongation factor 2
IF: intermediate filament
MAP: microtubule-associated protein
MT: microtubule
PTM: post-translational modification
TAP: tubulin-associated protein
